# Neural Mechanisms Underlying Breathing Complexity

**DOI:** 10.1371/journal.pone.0075740

**Published:** 2013-10-03

**Authors:** Agathe Hess, Lianchun Yu, Isabelle Klein, Marine De Mazancourt, Gilles Jebrak, Hervé Mal, Olivier Brugière, Michel Fournier, Maurice Courbage, Gaelle Dauriat, Elisabeth Schouman-Clayes, Christine Clerici, Laurence Mangin

**Affiliations:** 1 Laboratoire Matière et Systèmes complexes, UMR 7057, CNRS, Université Paris 7, Paris, France; 2 Service de Radiologie, APHP, Hôpital Bichat-Claude Bernard, Paris, France; 3 Institute of Theoretical Physics, Lanzhou University, Lanzhou, China; 4 Unité Inserm 698, Université Paris 7, Paris, France; 5 Ecole Normale Supérieure, Paris, France; 6 Service de Pneumologie B, APHP, Hôpital Bichat-Claude Bernard, Paris, France; 7 Département de Physiologie-Explorations fonctionnelles, APHP, Hôpital Bichat-Claude Bernard, Paris, France; 8 Unité Inserm 700, Université Paris 7, Paris, France; 9 Centre d’Investigation Clinique APHP, Hôpital Bichat, Paris, France; Clinica Universidad de Navarra, Spain

## Abstract

Breathing is maintained and controlled by a network of automatic neurons in the brainstem that generate respiratory rhythm and receive regulatory inputs. Breathing complexity therefore arises from respiratory central pattern generators modulated by peripheral and supra-spinal inputs. Very little is known on the brainstem neural substrates underlying breathing complexity in humans. We used both experimental and theoretical approaches to decipher these mechanisms in healthy humans and patients with chronic obstructive pulmonary disease (COPD). COPD is the most frequent chronic lung disease in the general population mainly due to tobacco smoke. In patients, airflow obstruction associated with hyperinflation and respiratory muscles weakness are key factors contributing to load-capacity imbalance and hence increased respiratory drive. Unexpectedly, we found that the patients breathed with a higher level of complexity during inspiration and expiration than controls. Using functional magnetic resonance imaging (fMRI), we scanned the brain of the participants to analyze the activity of two small regions involved in respiratory rhythmogenesis, the rostral ventro-lateral (VL) medulla (pre-Bötzinger complex) and the caudal VL pons (parafacial group). fMRI revealed in controls higher activity of the VL medulla suggesting active inspiration, while in patients higher activity of the VL pons suggesting active expiration. COPD patients reactivate the parafacial to sustain ventilation. These findings may be involved in the onset of respiratory failure when the neural network becomes overwhelmed by respiratory overload We show that central neural activity correlates with airflow complexity in healthy subjects and COPD patients, at rest and during inspiratory loading. We finally used a theoretical approach of respiratory rhythmogenesis that reproduces the kernel activity of neurons involved in the automatic breathing. The model reveals how a chaotic activity in neurons can contribute to chaos in airflow and reproduces key experimental fMRI findings.

## Introduction

Complexity is a universal phenomenon widely described in physics as well as in living organisms in biology and physiology. In the human brain, neural networks are complex [1] and communication between neurons occurs through a wild variety of codes such as bursting oscillations, which is a brief epoch of rapid firing. Such bursting behavior of the neuron oscillations may exhibit nonlinear deterministic chaos [2]. The human respiratory system displays several level of complexity: the bronchial tree has a fractal structure with various degrees of self-similarity and the airflow dynamics inside exhibits chaos during rhythmic breathing [3]. Why rhythmic breathing generates chaos in human airflow remains unknown. Breathing is maintained and controlled by a network of neurons in the brainstem that generate respiratory rhythm while receiving regulatory inputs. Pace-maker like neurons generating rhythmic breathing have been identified in 2 brainstem regions in rodents, one located in the rostral ventro-lateral (VL) medulla, the pre-Bötzinger complex [4]–[8], and the other close to this region, the parafacial respiratory group [9]–[13]. Recent evidence suggests that both groups of neurons are coupled oscillators that work in tandem to synchronize respiratory rhythm [9], [10], [13]. Moreover, these automatic neuronal groups have two important properties: they are capable of different synchronization regimes depending on the level of their excitabilities [13] and their dynamics exhibit chaotic spike-bursting oscillations in some circumstances [14]. Indeed, neural population activity recorded locally in the pre-Bötzinger complex of neonatal rat brainstem slices exhibit chaotic dynamics, when neuronal excitability is progressively elevated [14]. This is a strong argument to hypothesize that the chaos-like complexity of airflow in humans is an intrinsic property of central respiratory generators. In addition, both respiratory rhythm and airflow control have common genetic determinants [15]. However, breathing is also modulated by the state of airways [16], by the chest wall [17], the lung, by chemical afferents sensitive to hypercapnia, hypoxia or acidosis [3] and by mechanical afferents from the airway, lung, chest wall, respiratory muscles as well as by supra-pontine commands. A previous study has shown that the structural and mechanical properties of the bronchial tree, lung and chest wall in humans are not sufficient to generate chaos in airflow in the absence of a central neural drive [18]. Nevertheless, it is still unclear in humans to what extent the complex dynamics of the respiratory center contributes to airflow complexity.

We used both experimental and theoretical approaches to decipher the brainstem neural substrates of ventilatory complexity in humans. Complexity of airflow was estimated during inspiration and expiration at rest, and during an inspiratory effort with resistive load, used as an indirect neural stimulus. Brainstem regions of interest of the respiratory pacemakers were located with fMRI [19] in the rostral ventro-lateral medulla containing the pre-Bötzinger complex, and in the caudal ventro-lateral pons containing the parafacial group. Our goal was to evidence brainstem neural correlates of airflow complexity. We also analyzed airflow in a disease state in patients with chronic obstructive pulmonary disease (COPD). COPD is the most frequent chronic lung disease in the general population and is mainly due to tobacco smoke. Patients with COPD have an impaired lung function with an increased respiratory load due to small airways obstruction by inflammation and remodeling. Lung parenchyma destruction or emphysema is often associated with distal obstruction. Airflow obstruction associated with hyperinflation and respiratory muscles weakness are key factors contributing to load-capacity imbalance and hence increased respiratory drive [20]. At the end stage of the disease, the patients have respiratory insufficiency with home oxygen therapy while the neural respiratory drive is extremely high. We hypothesized that chaos in airflow should be altered in COPD patients but that such alterations should still correlate with the activity of the brainstem respiratory centers. Further, we developed a mathematical model of respiratory rhythmogenesis to reproduce the basic activity modes of neurons involved in the automatic breathing in healthy subjects and COPD patients. The model therefore reveals how a chaotic activity in neurons can contribute to chaos in airflow and reproduces key experimental fMRI findings.

## Results

**Figure 1:**
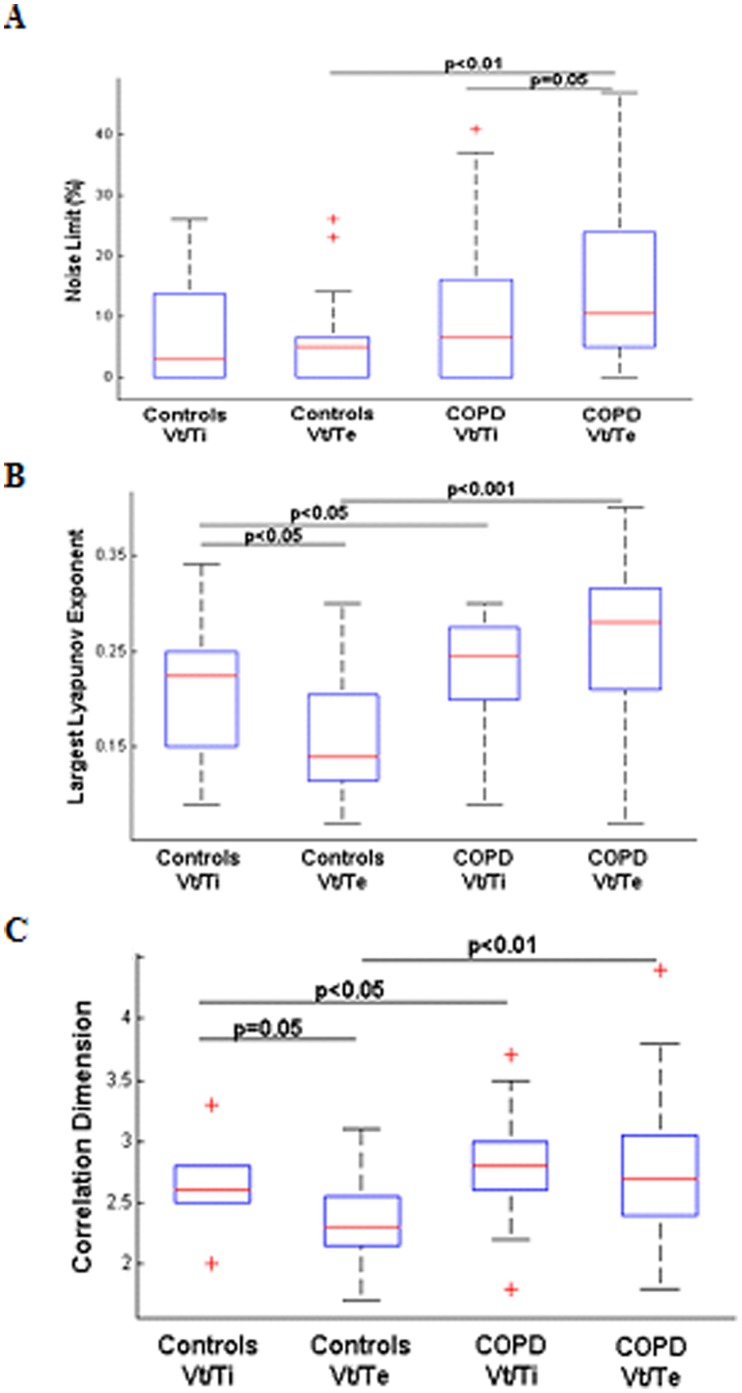
Chaos characterization of airflow during inspiration (Vt/Ti) and expiration (Vt/Te) in the controls and COPD patients. A: Noise Limit value (%), B: largest Lyapunov exponent, C: correlation dimension. The boxes encompass the interquartile range with indication of the median, the whiskers delimit the 95^th^ percentile of the data distribution. Paired and unpaired Ttest.

The characteristics of the whole population, healthy subjects and patients with chronic obstructive pulmonary disease (COPD), are shown in Tables 1 and S1. No difference was noted in end-tidal P_CO2_ (P_ETCO2_) measurements between healthy subjects and COPD patients either during resistive load or during resting state fMRI (Figure 1).

**Table 1:** Characteristics of the participants.

	Controls (n = 25)	COPD(n = 25)	pvalue
**Age (yr)**	52±11	56±9	*p = NS*
**Gender (M/F)**	14/11	14/11	*p = NS*
**Height (m)**	1.71±0.09	1.71±0.25	*p = NS*
**Weight (Kg)**	69±15	67±15	*p = NS*
**Body mass Index**	23±3	23±3	*p = NS*
**FEV1/FVC (% predicted)**	79±5	44±14	*p<0.001*
**FEV1 (% predicted)**	106±11	52±22	*p<0.001*
**RV (%predicted)**	103±17	182±56	*p<0.001*
**TLC (%predicted)**	110±13	122±22	*p<0.001*
**PaO2 (kPa)**	12.3±1	10±1.4	*p<0.001*
**PaCO2 (kPa)**	5.3±0.4	5±0.5	*p = NS*
**Dyspnea at rest** **(Borg scale)**	0	3.5±1	*p<0.001*

*Values are mean ± SD. Pulmonary function estimate: FEV1/FVC forced expiratory volume in one sec/forced vital capacity; FEV1: forced expiratory volume in one sec; RV: residual volume; TLC: total lung capacity.*

### Chaos in Airflow during Inspiration is Higher than during Expiration in Healthy Subjects

#### Linear and nonlinear measurements of the airflow

The linear estimates (coefficient of variation (CV) and autocorrelation coefficient (AC)) of the airflow during inspiration and expiration are shown in Table S2. In the 25 healthy subjects, inspiratory flow yields higher variability (p<0.001) and lower value of the AC (p<0.001) than expiratory flow during unloaded breathing.

**Figure 2:**
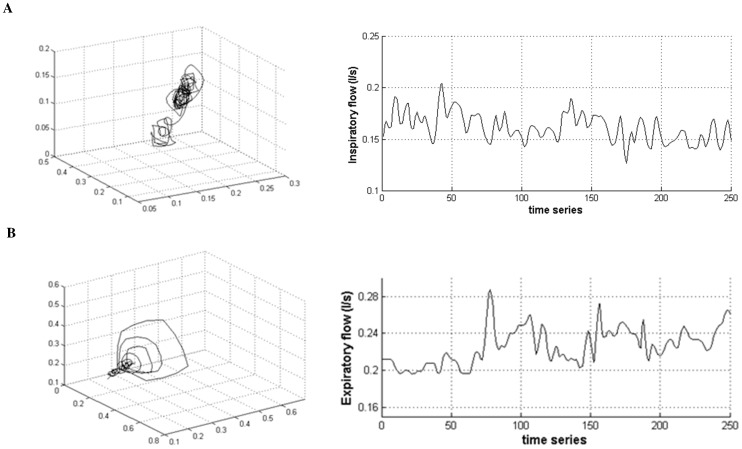
The chaotic signatures of the airflow in one healthy subject and one COPD patient are evidenced. The reconstructed attractors in the phase plane are shown on the left panel for one healthy subject during inspiration (A) and for one COPD patient during expiration (B). The corresponding time series are shown on the right panel.

The number of time series that exhibits a positive noise limit value characterizing chaos in airflow is equivalent for inspiration and expiration (Table S3). In the time series with positive noise limit, chaos in airflow is increased during inspiration as compared with expiration (largest Lyapunov exponent (LLE) and the correlation dimension (CD), p<0.05) (Figure 1). The attractor of the airflow is reconstructed in the phase plane during inspiration with the corresponding time series in one healthy subject (Figure 2A).

#### Cerebral fMRI results

**Figure 3:**
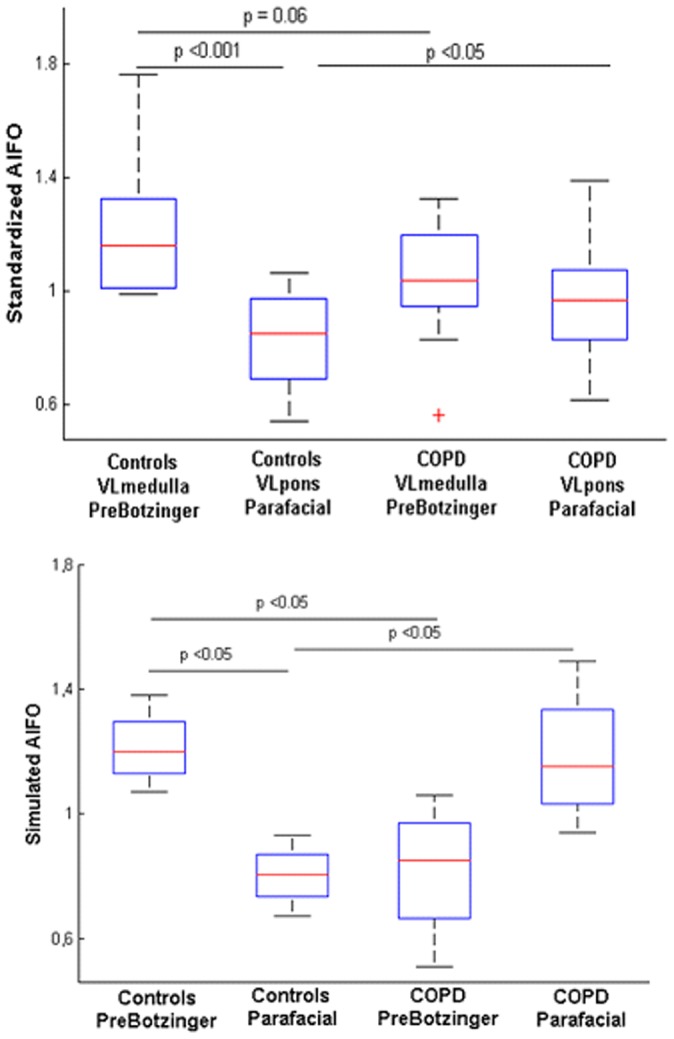
fMRI results of the brainstem respiratory centers at rest. Top. Amplitude of the low frequency oscillations (AlFO) of the resting state BOLD signal computed in controls and COPD patients. In healthy subjects the AlFO of the rostral ventro-lateral (VL) medulla that contains the pre-Bötzinger complex is higher than the VL medulla of the patients. Conversely, the ALFO of the caudal (VL) pons, which contains the parafacial respiratory group is higher in patients than the VL pons of healthy subjects. Bottom. Simulated AlFO obtained after hemodynamic convolution of the theoretical neural states. For controls, the chosen network scheme is described in Figure 8B, while for COPD patients, the network scheme is described in Figure 8C. Of note the synchronization regime describe in Figure 8D gave the same results as 8C for the simulated AlFO of the BOLD signal.

**Figure 4:**
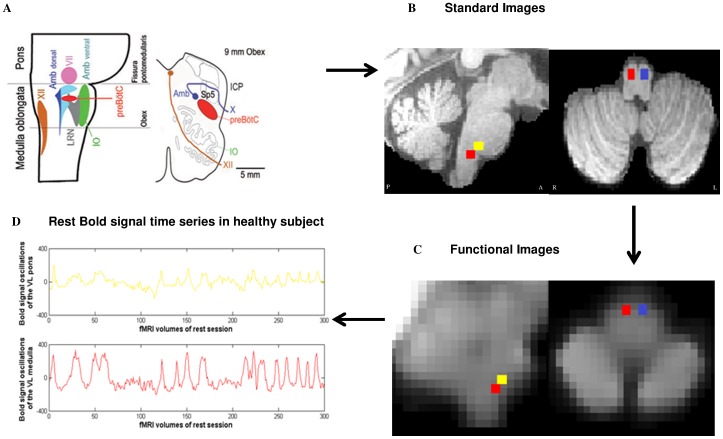
Localization on fMRI images of the regions of interest, the rostral ventro-lateral (VL) medulla with the pre-Bötzinger complex and the caudal VL pons with the parafacial. The regions of interest (brain mask with 4 cubes) were computed based on the recent article of Schwarzacher et al. (A) on individual standard images (sagittal and axial slices in B). Then the coordinates of the regions of interest were transformed from standard space to functional space (sagittal and axial slices in C). Two regions of interest of the right VL medulla and pons are shown in the sagittal slice (red and yellow) while two regions of interest of the VL medulla (red and blue) are shown on the axial slice. The region of interest of the left VL pons is not shown. Finally the mean time series were extracted for subsequent analyses (D). The corresponding mean time series of the VL medulla and VL pons are shown after extraction from the functional images, preprocessing analyses and regressing out with physiological covariates. The oscillations of the fMRI BOLD signal of the medulla in healthy subject (time series in red) are higher than those of the pons (time series in yellow). A: anterior, P: posterior, R: right, L: left.

In healthy subjects, we found that neural activity assessed in terms of the amplitude of low frequency oscillations (AlFO) of the BOLD signal located in the VL medulla is significantly higher than neural activity of the VL pons (p<0.001, n = 16) (Figure 3top, Figure 4). In COPD patients, the AlFO of the BOLD signal located in the VL pons, which contains the parafacial group, is significantly higher than the ALFO of the VL pons of healthy subjects (p<0.001, n = 16) (Figure 3top).

### COPD Patients Breathe with a Higher Level of Complexity during Expiration than Healthy Subjects

#### Linear and nonlinear measurements of the airflow

The linear estimates (CV and AC) of the airflow during inspiration and expiration are shown in Table S2. In the 25 patients with COPD, expiratory flow yields higher variability (p = 0.06) and AC (p<0.001) than inspiratory flow during unloaded breathing. The number of time series that exhibits a positive noise limit value characterizing chaos in airflow is equivalent for inspiration and expiration in COPD patients (Table S3). However, the number of chaotic time series during expiration is higher in COPD patients than healthy subjects (p = 0.001, Table S3). The attractor of the airflow is reconstructed in the phase plane during expiration with the corresponding time series in one COPD patient (Figure 2B).

In the time series with positive noise limit, chaos in airflow is increased during expiration as compared with inspiration (NL values, p = 0.05, Figure 1A). Moreover, as compared with controls, the levels of airflow complexity of expiration (NL value, p<0.001; LLE p<0.001; CD, p<0.01) as well as inspiration (LLE, p<0.05; CD, p<0.05) is higher (Figure 1B–C).

COPD patients having hypoxia (n = 10) do not exhibit differences from those being normoxic (n = 15) in terms of ventilatory complexity (noise limit value, largest Lyapunov exponent and correlation dimension). Furthermore, when comparing the chaotic indexes in the control group (n = 25) and in the COPD patients being normoxic (n = 15), (P_ETCO2_ being equivalent for both group), significant differences are evidenced with the noise limit value (NL controls: 5±7, NL COPD: 13±12 p<001) and the largest Lyapunov exponent (LLE controls: 0.15±0.08, LLE COPD: 0.27±0.1, p<0.001) of the expiratory flow.

Besides, COPD patients that exhibit severe dyspnea (Borg scale) have a significant higher level of expiratory flow chaos (correlation dimension) and AlFO of the VL pons than those with moderate and mild dyspnea (Figure S2).

#### Airflow complexity correlates with cerebral fMRI BOLD signal

**Figure 5:**
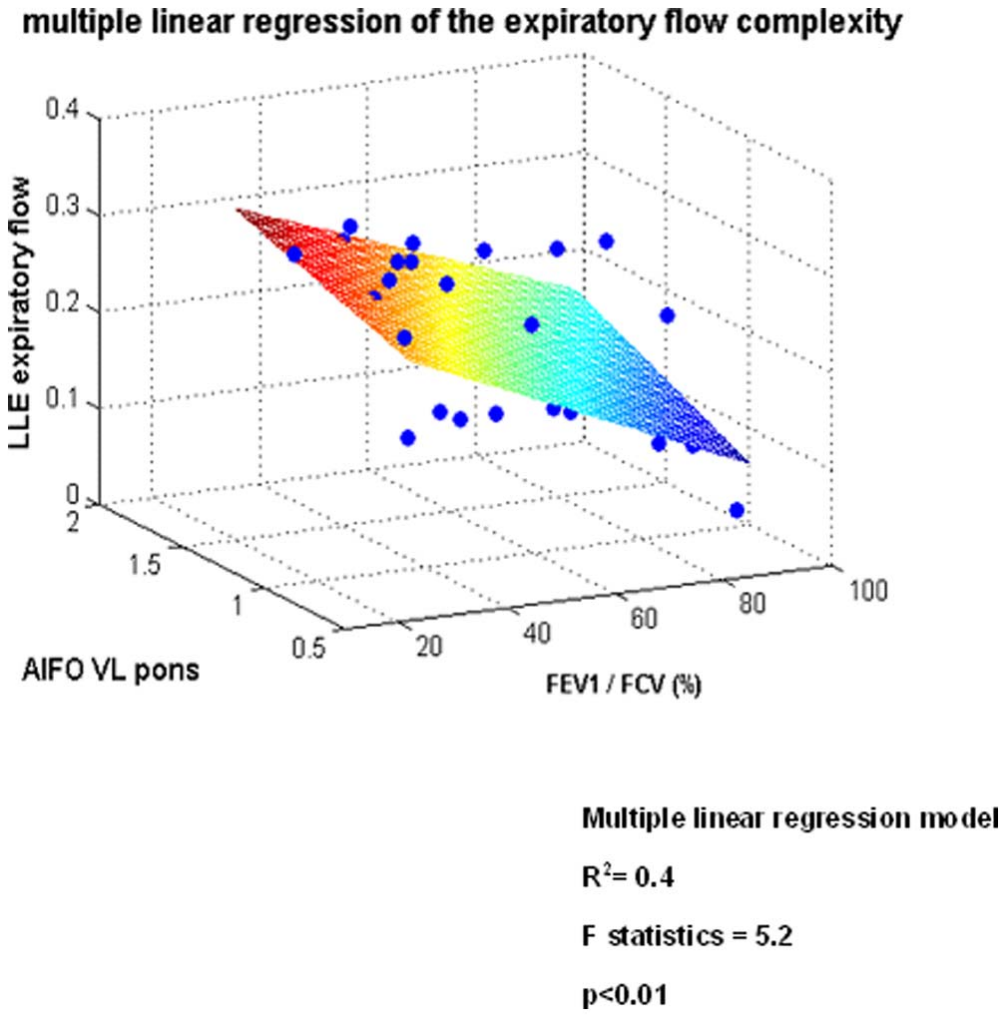
Central neural correlates of airflow dynamics in the whole population using multiple linear regression. Both the amplitude of the low frequency oscillations (ALFO) and the pulmonary function index FEV1/FVC significantly predict airflow complexity in the whole population: the lower the pulmonary function, the higher the value of the AlFO of ventro-lateral pons and the higher the complexity of expiration.

Univariate analysis in the whole population shows that the NL and the LLE values of the expiratory flow both positively correlates with AlFO of the VL pons (R^2^ = 0.4, p = 0.05 and R^2^ = 0.5, p = 0.04 for the NL and LLE respectively), the higher the complexity of expiration, the higher the neural activity of the VL pons. There is also an inverse relationship between the NL and the LLE of the expiratory flow and the pulmonary function index FEV1/FVC in the whole population (R^2^ = 0.45, p<0.05; R^2^ = 0.5, p<0.05, respectively, Figure S3). In healthy subjects, the chaotic level (NL) of airflow during inspiration strongly correlates with the neural activity of the VL medulla (R^2^ = 0.75, p = 0.01). In COPD patients, chaos (NL) during expiration correlates with the neural activity of VL pons (R^2^ = 0.4, p = 0.03). No correlation was evidenced between complexity of airflow and oxygen or carbon dioxide arterial pressures (PaO_2_, PaCO_2_). Multivariate analysis in the whole population showed that both neural activity of the VL pons and pulmonary function FEV1/FCV significantly predict the chaos of expiration (R^2^ = 0.4, F = 5.2 with p<0.01): the lower the pulmonary function, the higher the neural activity of the VL pons, the higher the chaotic level of expiration (Figure 5).

#### Airflow complexity and cerebral fMRI results during inspiratory load

**Figure 6:**
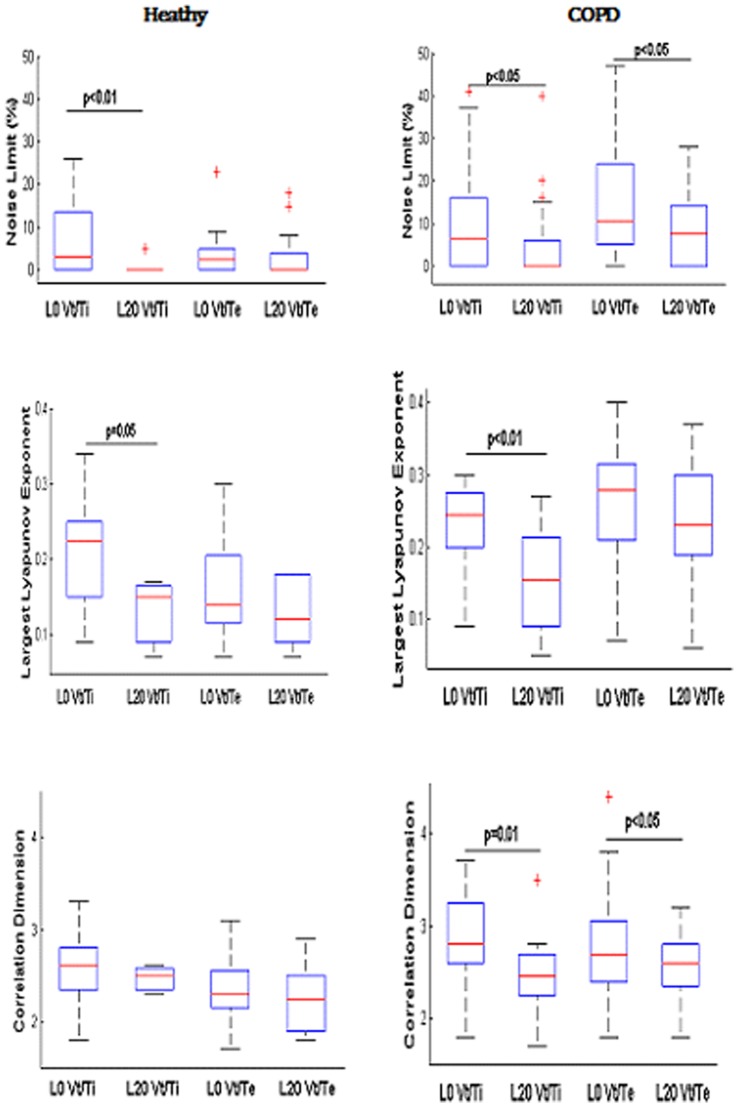
Loading inspiration leads to a diminution of complexity in airflow during inspiration in healthy subjects and during both inspiration and expiration and COPD patients. Noise Limit value, largest Lyapunov exponent and correlation dimension values are given from top to bottom. Lo no inspiratory load; L20: loading inspiration with 20 cmH2O/L/sec. The boxes encompass the interquartile range with indication of the median, the whiskers delimit the 95^th^ percentile of the data distribution. Paired and unpaired Ttests.

**Figure 7:**
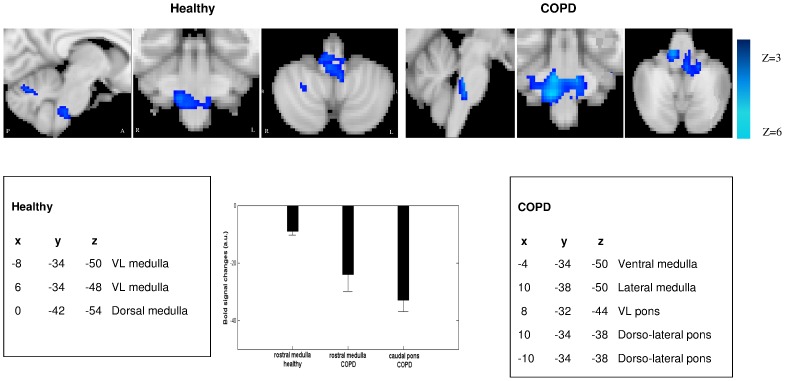
Negative BOLD signal of the respiratory brainstem network during inspiratory resistive loading in healthy subjects and COPD patients. Group analysis of healthy subjects (n = 16, left) and COPD patients (n = 17, right). Sagittal, coronal and axial slices are shown. In controls, negative BOLD signal is mainly evidenced in the ventro-lateral (VL) and dorsal medulla. In COPD patients, inhibition is located in the caudal lateral and dorsal pons, and in the lateral rostral medulla (color code in blue). A: anterior, P: posterior, R: right, L: left. Histograms showing the corresponding BOLD signal changes in the rostral medulla and caudal pons for controls and patients. C. The main coordinates (x,y,z) of the clusters that exhibit inhibitory BOLD signal are given in MNI space (Montreal neurological Institute).

Loading inspiration significantly increases the variability of the inspiratory flow, and the AC of the inspiratory as well as expiratory flows in both healthy subjects and patients with COPD (Table S2). In healthy subjects, inspiratory resistance significantly reduces airflow complexity during inspiration (Figure 6). Interestingly in COPD patients, loading inspiration leads to a diminution of complexity of inspiration (NL, LLE, CD) as well as expiration (NL, CD). Of note, loading inspiration did not change the P_ETCO2_ (Figure S1) and saturation of both populations. Loading inspiration in healthy subjects and COPD patients also leads to a diminution of fMRI BOLD responses in the VL medulla (healthy subjects and COPD) and pons (COPD) (Figures 7 and S4). During inspiratory loading in the whole population, the mean negative BOLD signal of the VL pons correlates with the CD of the expiratory flow (R^2^ = 0.7, p<0.01) while the mean negative BOLD signal of the VL medulla correlates with the LLE of the inspiratory flow (R^2^ = 0.6, p = 0.05).

Of note, healthy subjects and COPD patients also exhibit positive BOLD signal in the activated brain regions known to be involved in the voluntary control of respiratory muscles, i.e. sensory-motor, premotor and supplementary motor cortex area (data not shown).

#### Comparison of the correlation dimension of the original time series with surrogates

The correlation dimensions of the 137 experimental time series were compared with 5 surrogates (685 simulated time series) that match each original signal. Those surrogates were computed after assigning random phase. Significant differences were obtained between the original data paired with the corresponding average correlation dimension values from the matching surrogate (p<0.01, Wilcoxon signed-rank test), reinforcing the nonlinear features of the inspiratory and expiratory flows time series.

### Mathematical Model of Respiratory Rhythmogenesis

**Figure 8:**
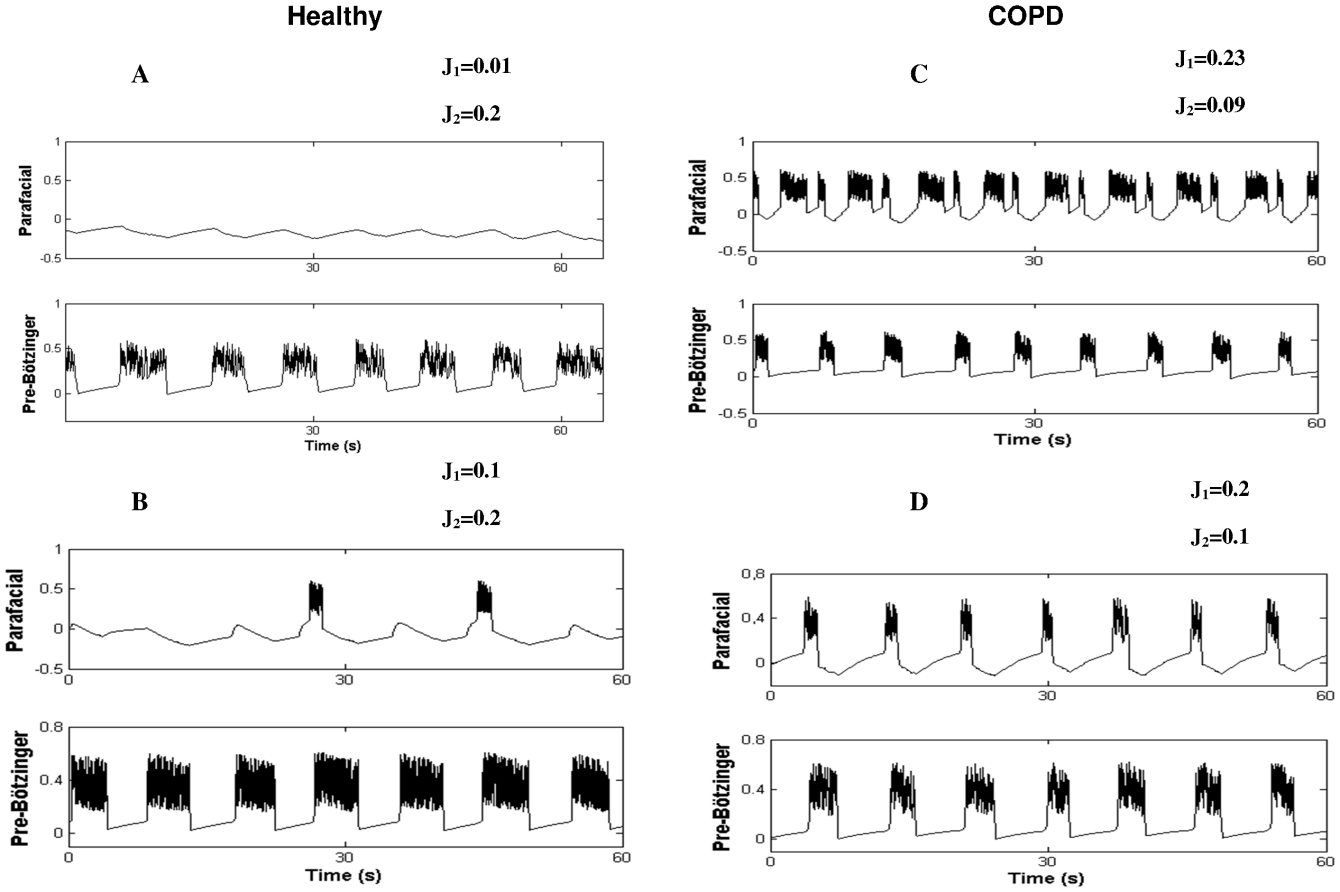
Simulations of different synchronization regimes in healthy subjects (A–B) and COPD patients (C–D) are depending on the excitability level of the parafacial repiratory group (J_1_) and the pre-Bötzinger complex (J_2_). Other fixed parameter values of the model are: ε  = 0.005, d = 0.4, β = 0.4, a = 0.2, m_0_ = 0.864, m_1_ = 0.65, δ = 0.2, x_th_ = −0.02 (threshold for calcium current), τ_1_ = 10 and τ_2_ = 2 for the parafacial while τ_1_ = 5, τ_2_ = 10 for the pre-Bötzinger. In COPD patients, the parafacial respiratory group of the brainstem has a higher excitability level than healthy subjects and drives the pre-Bötzinger (active inspiration and expiration). (See results for comments).

**Figure 9:**
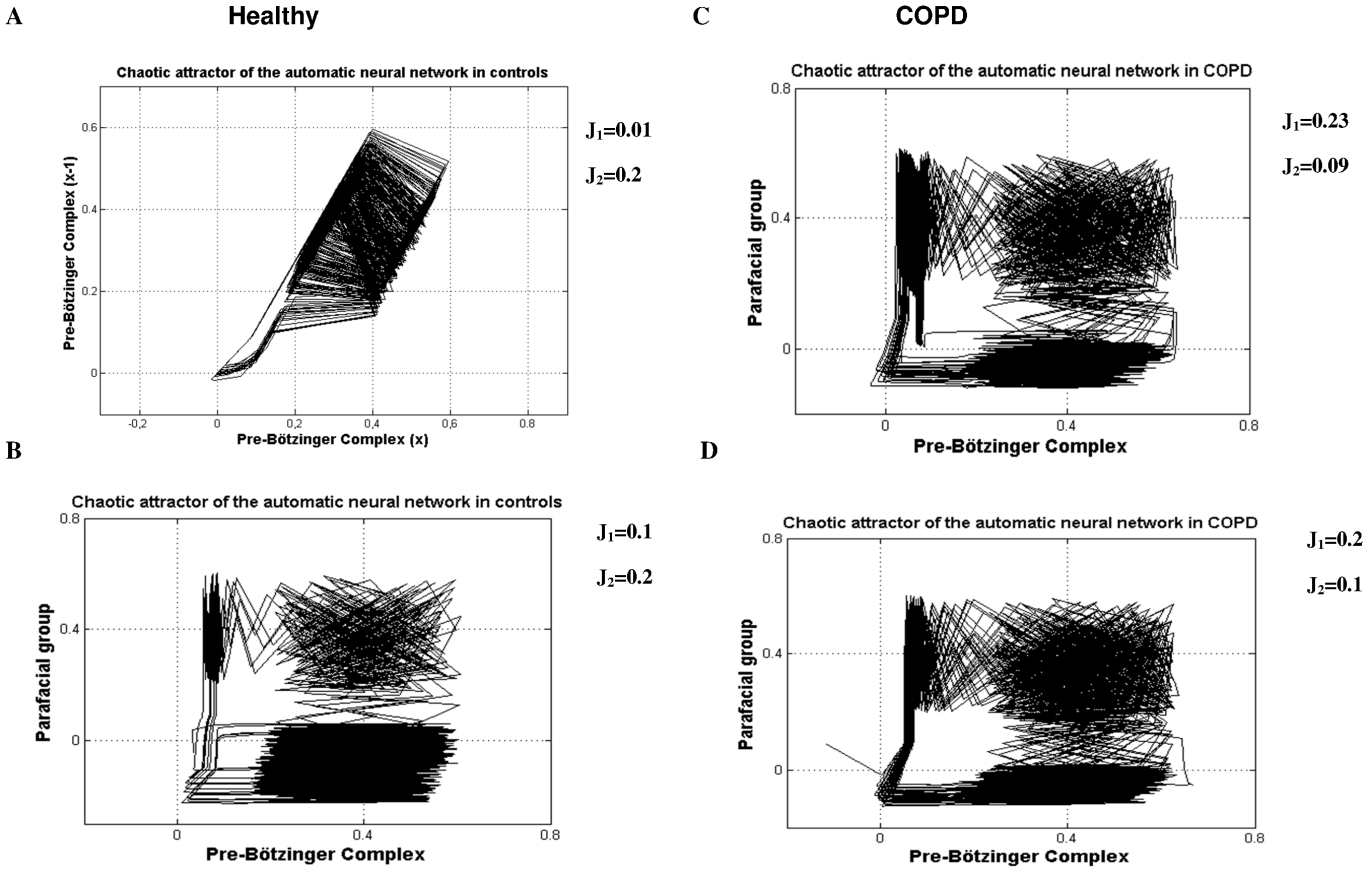
Chaotic attractor of the 2 synchronized pacemakers for respiratory rhythmogenesis in healthy subjects (A–B) and COPD patients (C–D) after simulations. Each attractor is given according to the different network regime presented in Figure 8. The figure reveals that the coupling between both neuronal pacemaker exhibit nonlinear deterministic chaos (9B–C–D).

The present model is the first attempt to reproduce respiratory rhythmogenesis in healthy humans and COPD patients with experimental data. The model considers two chaotic pacemakers, the inspiratory (Pre-Bötzinger) and expiratory (parafacial) generators that work together via chemical synaptic connection, either activated or inhibited, to synchronize the respiratory cycle. Different dynamics are evidenced depending on the excitability level of the neurons. In the model, the parameters J_1_ and J_2_ represent the excitability level of the parafacial and pre-Bötzinger respectively. Experimental results show that healthy subjects display more complexity during inspiration than expiration and that the low frequency oscillations of the BOLD signal located in the rostral VL medulla have higher amplitude than oscillations of the caudal VL pons. From this, we postulate that the pre-Bötzinger complex is highly likely more excitable than the parafacial group, and drives the respiratory rhythm (active inspiration). Simulation of this network scheme is shown in Figure 8 with two possible regimes depending on the parameter values J_1_ and J_2_. In the first regime (Figure 8A), the parafacial has a very low excitability and is entirely depressed with no action potential. This network scheme is similar to the one described in adult rats, the “no-handshake process” [13]. The corresponding attractor of this scheme entirely relies on the pre-Bötzinger dynamics (Figure 9A). In the second regime (Figure 8B), while the pre-Bötzinger is the dominant pacemaker still driving the respiratory cycle, the parafacial group is occasionally relieved by specific physiological conditions [21]. Experimental results show in COPD patients that airflow complexity is higher during expiration than inspiration and that the low frequency oscillations of the BOLD signal located in the VL pons have higher amplitude than the oscillations of the VL pons of healthy subjects. In patients, we therefore hypothesize that the expiratory neurons located in the VL pons are more excitable than the pre-Bötzinger and drive the respiratory cycle. In this network scheme (Figure 8C), the more excitable parafacial group triggers the pre-Bötzinger which in turn inhibits the parafacial with a post-inhibitory rebound burst. The parafacial then switch-off inspiration. This network scheme is similar to the “full-handshake process” described in neonatal rats [13]. The corresponding attractor of this synchronization process mainly relies on the parafacial neurons dynamics (Figure 9C). Another synchronization regime may coexist in the disease state, when the excitability level of the expiratory group is slightly lower: the “half-handshake” process in which the parafacial still triggers the pre-Bötzinger which in turn induces a delayed post-inhibitory rebound burst that triggers a new pre-Bötzinger activation (Figures 8D and 9D).

#### Modeling fMRI signal based on simulated neural activity in healthy subjects and COPD patients

To confirm our hypotheses on respiratory rhythmogenesis in healthy subjects and COPD patients, we performed 5 runs of simulations (250 action potentials with chaotic bursting oscillations for both pacemakers) of the synchronization regimes shown in Figure 8B–C. fMRI signals can then be modeled as a result of the convolution of the obtained neural states with a hemodynamic response function and added noise (see methods). The amplitude of the low frequency oscillations of the fMRI signal is then computed and shown in Figure 3 (bottom). The model is able to replicate the experimental fMRI results in both healthy subjects and COPD patients.

## Discussion

We are the first, to our knowledge, to identify and describe the brainstem neural substrates underlying breathing complexity in healthy humans and patients with lung disease. fMRI scans revealed neural activity in the rostral ventro-lateral medulla and caudal ventro-lateral pons fitting the neural dynamics of respiratory rhythmogenesis. We then provided evidence that these central neural activities significantly correlate with the dynamical characteristics of the inspiratory and expiratory airflow in healthy humans and COPD patients (Table S4). Further, we developed a mathematical model of chaotic pacemakers where different neuronal excitabilities entrain different synchronization regimes and complexities that replicate key fMRI findings in humans.

### Source of Human Ventilatory Complexity

We decided to focus on the core automatic network generating respiratory rhythmogenesis [4], [5], [9]–[12], [22], [23] since previous experimental and clinical works highlighted its potential contribution to airway flow complexity [14], [18], [24]. In the present study, ventilatory complexity significantly correlates with the activity of the respiratory central pattern generators assess with cerebral fMRI: in COPD patients, the increase in airflow complexity during expiration comes along with the higher VL pons parafacial activity while healthy subjects exhibit higher VL medulla activity with greater complexity during inspiration. Such parallel changes underline the contribution of the respiratory pacemaker neurons in airflow complexity. Previous works analyzed the mechanisms modulating chaos in airflow but failed to decipher the brainstem neural contribution to airflow complexity in human. It was previously shown that mechanical loading conditions alter chaos with an increase complexity in circumstances improving the load capacity-balance of the respiratory system [25], that breathing complexity was impaired during carotid stenosis due to the effects of autonomic baroreflex impairment on breathing control [26], and finally that chemoreceptor stimulation of ventilation by hypercapnia led to a high level of complexity [3]. Interestingly, while Fiamma et al. [3] showed in one study that hypercapnia stimulated ventilation and increased airway flow chaos, Pattinson et al. [27] demonstrated in a neuroimaging work that carbon dioxide stimulus activates brainstem respiratory centers of the ventral pons, rostral pons and lateral medulla. Some of these activated area overlapped with our regions of interest during the block design paradigm. Besides, we used a theoretical approach of respiratory rhythmogenesis to reproduce the core activity modes of neurons involved in the automatic respiratory network scheme in humans with two synchronized chaotic pacemakers, one driving inspiration, the pre-Bötzinger complex and the other driving expiration, the parafacial group. We chose to develop a map-based model [28], [29] for its relative simplicity compared with Hodgkin-Huxley formalism, and for its ability to generate spontaneous chaotic bursting activity. The model was further refined to incorporate post-inhibitory rebound bursting behavior. The mathematical model we propose is in line with previous experimental and theoretical works [13], [14]. In addition, it is able to exhibit chaotic behavior depending on the parameter value J which is the excitability level of the neuron. Above all, it reveals how a chaotic activity in neurons (Figure 9) contributes to chaos in airflow (Figure 2). Through controlling the excitability levels of the pre-Bötzinger and parafacial neurons in the mathematical model, different synchronizations and level of complexity appear. The choice of the parameter values, among them J_1_ and J_2,_ are motivated by 2 characteristics: the ability to exhibit chaotic spike bursting oscillations (J value between Jmin and Jmax) and the specific synchronization regimes. Finally we verified our hypotheses on respiratory rhythmogenesis in healthy human and COPD patients (re-activation of the parafacial) with the mathematical model of the full handshake process and we were able to mimic the experimentally fMRI signals of the brainstem ventro-lateral medulla and ventro-lateral pons (Figure 3).

### COPD Patients Breathe with a Higher Level of Complexity during Expiration than Controls

We found that patients with chronic obstructive pulmonary disease breathe with a higher level of complexity in airflow than healthy subjects. These unexpected findings cast doubt on the traditional view that complexity systematically degrades in disease state [30], [31]. Inspiratory and expiratory complexity changes parallel the activity of the VL medulla and VL pons, which contains the pre-Bötzinger and parafacial neurons respectively. It is therefore an in vivo estimate of the respiratory center function in humans as previously shown [18]. In healthy subjects, airflow complexity is higher during inspiration than expiration thus reflecting active inspiration while expiration is usually passive due to the elastic recoil of the lung. Conversely, patients with COPD have a higher level of complexity during expiration as compared with healthy subjects because they actively expire. In patients, fMRI revealed greater neuronal activity in the caudal VL pons region than in healthy subjects. Further studies are required to elucidate if patients having a high excitability of the caudal VL pons with the parafacial group, are those who effectively actively recruit their expiratory muscles, as suggested by Yan et al. [32]. We show that the excitability level of the neurons involved in respiratory rhythmogenesis in humans may vary depending on the physio-pathological conditions. These findings are in agreement with previous experiments in rats. In neonates, the parafacial expiratory group which has a high excitability level is dominant and drives the pre-Bötzinger [9], [10], [13], while in adults animals the parafacial is normally depressed and the pre-Bötzinger becomes dominant [5], [9], [10], [15]. Direct stimulation of parafacial neurons has been recently shown to promote active expiration in adult rats [33]. It is also possible to reactivate [34] the parafacial group during hypoxia [35]. Moreover, a previous study demonstrated that patients passively driven by a mechanical ventilator do not exhibit complexity in airflow whereas those with signs of active expiratory control displayed an increase complexity [18]. COPD patients have a forced expiratory flow limitation, which promotes the recruitment of abdominal muscles to sustain ventilation. The expiratory oscillator is probably turned on in patients to sustain ventilation in response to the increased respiratory load and hypoxia. Healthy subjects and COPD patients do differ in terms of PaO_2_. However, the contribution of O_2_ sensitive-chemoreceptors to the increase in airflow complexity in patients is weak since no difference between normoxic and hypoxic COPD patients is evidenced. Moreover, expiratory flow complexity differs between controls and normoxic COPD patients. From these results, we postulate that mechanical abnormalities due to disordered lung mechanics play a critical role in subsequent complexity alterations. Indeed, we found correlations between decrease pulmonary function and chaotic components in both univariate (Figure S3) and multivariate analyses (Figure 5). The increase in airflow complexity in patients is also related to systemic inflammation as shown during COPD [36]. A previous work in rats showed that brainstem cytokine level is high in a model of acute respiratory failure and this was strongly related to the increase in ventilatory complexity [24]. Finally, one additional explanation relies on the pathological narrowing of the bronchial tree and the direct “physical” consequences on the airflow: it is possible that some airflow turbulence due to local structural abnormalities and disordered lung mechanics directly contributes to increase airflow chaos, especially during expiration.

Interestingly, we could discriminate COPD patients with mild, moderate and severe dyspnea at rest according to expiratory flow complexity and the neural activity of the VL pons: patients with a severe dyspnea had a higher level of expiratory flow complexity and greater activity of the VL pons, as compared with patients having mild dyspnea. This difference was even less sensitive for the pulmonary function (Figure S2). Therefore, COPD patients having a severe dyspnea unexplained by a worsening of their pulmonary function, may exhibit an altered neuronal excitability of the VL pons, thereby reinforcing the central determinism of dyspnea.

### Chaos in Airflow Decreases during Inspiratory Load, While Neural Activity of the Respiratory Centers Yields Negative BOLD Signals

Loading inspiration reduces airflow complexity with a parallel inhibition of the BOLD signal in the rostral medulla of healthy subjects. Our results differ from a previous study in 8 healthy subjects that did not find any effect of inspiratory loading on airflow chaos [37]. Differences in the experimental protocol may explain these discrepancies, i.e. the number of subjects included (25 healthy subjects in our study) and the duration of the load applied (15 minutes in our protocol). Furthermore, a previous work using fMRI found activation in the ventral pons of healthy subjects during inspiratory loading [38]. We point out that in the study of Gozal et al. [38] the protocol was different in terms of the load applied (30 cmH_2_0/L/sec in their study), fMRI image acquisition and processing, specifically for the inclusion of confounding statistical regressors in the model. Moreover, negative BOLD signal changes were not specifically investigated [39]. Besides, it has been shown in 6 healthy subjects that voluntary hyperpnea targets the superior dorsal medulla of the brainstem [40]. In our study, the dorsal medulla showed significant de-activation during inspiratory resistive load. Differences in the stimulus applied (resistive load in our protocol) and in the characteristics of the healthy controls (16 controls in our study with older mean age 52±11) may explain these discrepancies. Of note, healthy subjects and COPD patients also exhibit activated brain regions known to be involved in the voluntary control of respiratory muscles, i.e. sensory-motor, premotor and supplementary motor cortex area (data not shown). The fact that the mechanical inspiratory load activates these cortical centers and de-activates in parallel the automatic network is physiologically relevant.

Loading inspiration in COPD patients leads to a diminution of airflow complexity of inspiration as well as expiration. These results are in line with the possible dual organization of respiratory rhythmogenesis in patients where reactivation of the parafacial occurred (Figure 8C–D). Once a stimulus is applied during inspiration it echoes on the other pacemaker due to the coupling characteristics. In addition, a diminution of fMRI BOLD responses in the two regions VL medulla and pons occurs in parallel in patients (Figure 7 and Table S3).

#### Study limitations

A major challenge in application of fMRI to respiratory studies is the limited spatial and temporal resolutions of the BOLD signal [41], making it difficult to pinpoint precisely the specific brainstem respiratory related structures, which are generally rather small and heterogeneous with time-varying respiration related fluctuations. The pre-Bötzinger complex is a small structure and is bordered by other respiratory related nuclei including the Bötzinger complex. The parafacial respiratory group is a spread-out structure and contains both expiratory-related neurons and chemosensory neurons. We are however confident with our fMRI measurements for three reasons: (i) the first reason relies on the neuroanatomical paper recently published from Schwarzacher et al. [6]. The authors accurately identify in human brain autopsy the location of the pre-Bötzinger complex. The diameter of the complex is around 5–6 mm, in the ventro-lateral region of the rostral medulla, 9 mm from obex, below Fissura Pontomedullaris. For all participants of our fMRI protocol, we individually computed these coordinates in standard images. Then the regions of interest were centered on these coordinates and transformed from standard space to functional space for the extraction of the time series. The parafacial respiratory group is located near the pre-Bötzinger in the caudal ventro-lateral pons, ventro-laterally to the facial nerve nucleus VII [6], [7], above Fissura Pontomedullaris. (ii) The second reason relies on the de-activation regions evidenced during the block design paradigm with inspiratory resistive load. Theses inhibited regions overlapped the coordinates defined in the rest fMRI acquisition and we also found strong correlation between the mean negative BOLD signal and the chaotic component using the same coordinates than rest fMRI acquisition. (iii) The third reason concerns the theoretical part of the work. We modeled respiratory rhythmogenesis with two pacemakers that synchronously handshake one another, depending on their excitability level [13]. The resulting neural time series of the pre-Bötzinger and parafacial groups, convolved with a hemodynamic function plus noise replicate experimental fMRI signal in healthy subjects and COPD patients.

Furthermore, we cannot exclude the potential influence of emotion via the limbic system on the automatic network [42]. However before airflow recordings begin, the subjects were allowed to adapt for 5 minutes to the materials and were quiet. We also removed the first 2 minutes of recordings for subsequent analyses. Additionally, we took time to explain the fMRI protocol to both healthy subjects and COPD patients. For fMRI protocol, the participants were instructed to ‘keep their eyes closed and think of nothing in particular’. They were instructed to refrain from cognitive, language, and motor tasks. The participants knew that a physician was near the scanning room and they all had the possibility to stop the images acquisition if a problem arised. We therefore minimized as much as possible the possible influence of emotions on our experiments.

#### Perspectives

In this study, we decipher the brainstem neural substrates of airflow complexity in humans. We also shed new lights on the brainstem neural control of respiratory muscles in patients with COPD. The patients have an increased complexity of the airflow during expiration that correlates with the high activity of VL pons. COPD patients reactivate the parafacial neuronal group, as shown with the mathematical model and fMRI results, to sustain ventilation. These findings may be involved in the onset of respiratory failure when the neural network becomes overwhelmed by respiratory overload as suggested by previous works [43], [44]. Future works analyzing the relationships between automatic and cortical network from a theoretical and experimental viewpoint will help to clarify the mechanisms preceding acute respiratory failure. Moreover, we show that COPD patients having a severe dyspnea unexplained by a worsening of their pulmonary function, may exhibit an altered neuronal excitability of the VL pons, thereby reinforcing the central determinism of dyspnea. Identifying the activity of the respiratory pacemakers through both airflow complexity and functional imaging techniques opens new strategies to refine COPD patient phenotypes.

## Methods

### Participants and Protocol

Stable patients (n = 25) with COPD (no exacerbation for 4 weeks) were recruited from the Physiology and Respiratory disease departments of the Bichat University Hospital 2011–2012. Inclusion criteria were patients above eighteen having mild to severe COPD according to clinical and pulmonary function test criteria [45]. Exclusion criteria were home oxygen therapy, neurological disease, past history of stroke, psychiatric disorder, body mass index above 30 kg/m^2^, contraindication to cerebral functional magnetic resonance imaging. After given written informed consent, patients had a clinical examination and pulmonary function tests. In COPD patients, dyspnea was quantified at rest using Borg scale. An age-matched control group (n = 25) was recruited from the Centre d’ Investigation Clinique of the Bichat Hospital. The protocol was approved by the ethics committee Ile-de-France 1.

Subjects were comfortably seated and were asked to keep their eyes open. They wore a nose clip and breathed through a mouthpiece connected to a low resistance pneumotachograph (MLT1000L-AD Intruments) via a two-way non-rebreathing valve (Hans Rudolph 1410 Series). Ventilatory flow, digitized at 400-Hz sampling rate was recorded on a PC computer in the form of data files for subsequent analysis (Chart5, AD Instruments). Mouth pressure was measured at the mouthpiece and connected to a pressure transducer (MLT0699-AD Instruments). Ventilatory flow and mouth pressure were synchronously recorded on the PC computer via the PowerLab 4/25 (AD Instruments). End-tidal P_CO2_ (P_ETCO2_), measured from a side port of the mouthpiece and finger oxygen saturation were connected to a portable Oxi-capnography (MD-660P Comdek) for continuous acquisition. Before recordings began, the subjects were allowed to adapt for 5 minutes to the materials and were quiet. Recordings were performed during 15 minutes at the same time of the day for all subjects. Two sets of measurements were performed in random order, one with subjects breathing spontaneously and one with subjects breathing during the continuous application of an inspiratory resistive load of 20 cmH_2_0/L/sec (7100R20 Hans Rudolph). Reproducibility of our measurements was previously tested [26]. Ventilatory flow recordings will be available upon request to the corresponding author.

### Linear and Nonlinear Analyses of Airflow

The first two minutes of recording were excluded from the analyses. Inspiratory (Vt/Ti) and expiratory (Vt/Te) flows were computed on a breath-by-breath basis during spontaneous breathing and during the inspiratory effort, i.e. during continuous application of the resistive load on the inspiratory phase of the respiratory cycle.

### Analysis of Ventilation in the Time Domain and Autocorrelation Analyses

Fluctuations of the inspiratory and expiratory flows were first evaluated through their coefficients of variation (the ratio of the standard deviation to the mean). Autocorrelation of the flows was computed at a lag of one breath. It estimated the amount of correlated linear part of the flow [18], [26], [46].

### Nonlinear Analyses

#### Chaos detection

The noise titration technique [47] was used on the inspiratory and expiratory flow time series. This method has already been proven its accuracy to evidence the chaotic nature of human ventilation [3], [18], [26], [46]. It involved the simulation of time series with linear and nonlinear polynomial autoregressive model (Volterra-Wiener series) [48]. The best linear and nonlinear models are chosen according to the minimal information theoretic criterion. The null hypothesis, a stochastic time series with linear dynamics, is rejected if the best nonlinear model provided a significant better fit to the data than the best linear model using parametric (F-test) statistics at the 1% significance level. Once nonlinear determinism is indicated, white noise of increasing standard deviation is added to the data until nonlinearity can no longer be detected, i.e. the nonlinearity is ‘neutralized’. The noise limit (NL) is calculated as the percent of signal power added as noise to ‘titrate’ the data to the point of neutrality. Typically, an average NL value is obtained by repeating the titration procedure 5 times. Under this scheme, chaos is indicated by NL>0, and the value of NL provides a relative measure of chaos intensity. Conversely, if NL = 0, then it may be inferred that the series either is not chaotic or the chaotic component is already neutralized by the background noise (noise floor) in the data. We then estimated the largest Lyapunov exponent and the correlation dimension of the time series having a positive noise limit value.

#### Sensitivity to initial conditions

Complex dynamical systems are sensitive to initial conditions, and exhibit an exponential divergence in the phase space. This can be quantified through the study of the Lyapunov exponents spectrum and the calculation of the largest Lyapunov exponent (λ_L_: LLE). Consider two points on two nearby trajectories in the phase space, and assume the distance between them to be d(0). After time t, if the distance between the two trajectories becomes d(t), then the average divergence (separation after time t) can be written as:
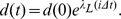
where λ*_L_* is the LLE of the system. In the present study, we used the algorithm proposed by Rosenstein et al. that has been shown to be particularly useful for small data series [49].

#### Irregularity

The correlation dimension is a fractal dimension reflecting the irregularity of the attractor of the system. It characterizes the “aperiodicity” of the system in the phase space. It is estimating by examining the scaling properties of the correlation sum [49]. From a time series 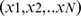, where N is the total number of points, the m dimensional vector in the phase space can be constructed by delay embedding:
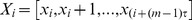
where, **τ** is the fixed time lag and m is the embedding dimension. Then the reconstructed trajectory of the actual dynamics can be written as 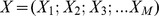 where 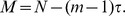


The correlation dimension can be calculated from the correlation integral of the time series. The correlation integral can be computed as follows [49], [50]:
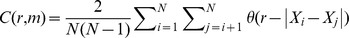
where, r is scale length, and θ is the Heaviside step function. Scaling of the function *C(r,m)* can be written as:



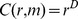



The correlation dimension (*D_corr_*) can be defined by
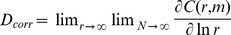
and for practical purpose, D_corr_ can be obtained from the slope of ln *C(r)* vs ln *r* plot.

Time lag was first estimated by a drop of the autocorrelation to 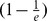 [49]–[51]. The optimal dimension was obtained after calculating the percentage of false nearest neighbors between points in phase space. A minimal number of false nearest neighbors was required [52]. The embedding dimension that adequately represents the system is the dimension that eliminates most of the false nearest neighbors allowing an adequate phase-space reconstruction of the underlying dynamic. An appropriate time lag and embedding dimension were estimated for each experimental time series.

#### Surrogate data

In order to test the nonlinearity that governs the dynamics, we have applied surrogate test [53]. First the Fourier transform of the original time series is computed. The phase is replaced by random numbers and finally the inverse Fourier transform is applied. Power spectrum is thus preserved although the nonlinear structures are destroyed [51], [53]. Correlation dimension was estimated for both the original data and five surrogates that match each original signal. A global test was carried out by a Wilcoxon signed-rank test comparing the correlation dimension values computed on the original data paired with the corresponding average correlation dimension values form the matching surrogate. Significant Wilcoxon rank test between the original and surrogates implies the nonlinear dynamics of the original data [18], [26], [46], [53].

### Cerebral Functional Magnetic Resonance Imaging

#### Protocol and image acquisition

Participants were imaged while lying comfortably in the scanner. Three sets of images were performed: structural, resting state and block design paradigm. For the structural and functional resting state, the participants breathed spontaneously while during the block design paradigm, they breathed via a mouthpiece connected a two-way non-rebreathing valve (Hans Rudolph 1410 Series) with nose clip. A small plastic tube of one meter length was connected to the inspiratory limb of the T-valve for application of the resistive load (20 cm/L/sec).

Physiological monitoring synchronized with the images acquisition was performed for the resting state and block design paradigm. Chest expansion was measured with a pneumatic belt and electrocardiogram was acquired with chest electrodes [54]. Sampling rates were 10 ms and 1 ms respectively. Respiratory volume per time (RVT) was computed from the respiratory waveform (chest belt) [55]. Maximum minus minimum of the waveform was divided by the breathing period for each breath cycle and then interpolated to the imaging repeat time (RT). P_ETCO2_ and saturation were also continuously recorded with 10 ms sampling rates. The RR cardiac interval, P_ETCO2_ and saturation (maximum values per breath), were also interpolated to the imaging RT.

Imaging was performed using a 3 Tesla MR scanner (General Electrics, USA) with a 64-channel head coil. T1-weight high resolution 3D volume covering the entire brain was acquired in controls (n = 16) and COPD patients (n = 17). Acquisition parameters were: 171 axial slices, 1.2 mm thickness with no gap echo time [Te] = 3.4 ms, repeat time [TR] = 8.6 ms, flip angle = 12°, matrix 256×256, field of view 240 mm×240 mm). The total acquisition time was 4 min 35 s.

T2-weighted echoplanar images were acquired for the resting state functional acquisition (52 axial slices, 4 mm thickness with no gap echo time [Te] = 19 ms, repeat time [TR] = 2000 ms, flip angle = 90°, matrix 64×64, field of view 240 mm×240 mm, and voxel dimension 3 mm^3^). Acquisition time was 10 min08 s, yielding 300 whole brain volume. For the resting state, the participants were instructed to ‘keep their eyes closed and think of nothing in particular’. They were instructed to refrain from cognitive, language, and motor tasks as much as possible, but not to fall asleep. Resting state fMRI scans will be available upon request to the corresponding author.

The second set of functional image was performed during a block design, which consists in 5 cycles of rest periods (36 sec), in alternate with active period (36 sec) during which the inspiratory resistive load (20 cmH_2_0/L/sec) was applied on the breathing circuit. MRI parameters were: 52 axial slices, 4 mm thickness with no gap echo time [Te] = 33 ms, repeat time [TR] = 3000 ms, flip angle = 90°, matrix 64×64, field of view 240 mm×240 mm, and voxel dimension 3 mm^3^. Total acquisition time was 6 min12 s, yielding 120 whole brain volumes.

#### Image analyses

Image processing was performed using FSL software (http://www.fmrib.ox.ac.uk/fsl, Oxford University).

#### Resting state fMRI

Preprocessing steps included motion correction using MCFLIRT [56] slice timing corrections, non-brain removal using BET [57], spatial smoothing using a Gaussian kernel of full-width-half-maximum 6 mm, multiplicative mean intensity normalization of the volume at each time point. A brain mask was constructed with four regions of interest (cubes radii 6 mm) individually positioned on standard images over the brainstem in regions known to cover the respiratory generator nuclei in rostral ventro-lateral medulla oblongata and caudal ventro-lateral pons according to Schwarzacher et al. (Figure 4). These regions of interests were then transformed from standard space to functional space and the mean BOLD signal time series were then extracted. For all participants, the respiratory volume per time (RVT), the RR cardiac interval, P_ETCO2_ [58], [59] and saturation were included in a multivariate regression linear model to account for significant influences on the BOLD signal. These covariates were then regress out.

Low frequency oscillations have been used in resting state fMRI in physiology and pathology to analyze the functional connectivity among brain regions [60]–[62]. Amplitude of the low frequency oscillations (AlFO) of the resulting BOLD signal time series is also a mean to assess neuronal activation with fMRI [63], [64]. BOLD time series were detrended and filtered between 0.01 and 0.08 Hz to remove the effects of very low-frequency drift and high-frequency noise. Fast Fourier transform (FFT) was applied and the power spectrum obtained. The average square root of the power spectral density was calculated across 0.01–0.08 Hz and this represents the AlFO. For normalization purposes, the AlFO of each regions of interest was divided by the global mean AlFO value of the whole brainstem. The standardized AlFO have a value about 1 and this procedure is analogous to that used in PET studies [65]. Finally the mean of the normalized AlFO of the 2 cubes of the medulla and the 2 cubes of the pons were averaged.

#### Block design fMRI

Preprocessing step were the same as resting state fMRI with an additional high pass temporal filtering (Gaussian-weighted least-squares straight line fitting with sigma = 36 s). At the single level analysis we used a general linear model. Confounding regressors that potentially altered cerebral blood flow (RVT, P_ETCO2_, RR cardiac interval, saturation) were included. Voxel-wise statistical analysis was extended to a second (group) level in a fixed-effects analysis. After analysis, statistical images were registered to high resolution structural and/or standard space images using FLIRT [66]. Registration from high resolution structural to standard was then further refined using FNIRT nonlinear registration.

#### Statistical analyses of airflow dynamics and fMRI data

Matlab R2011a was used for statistical and signal processing analyses (Mathworks USA). Comparisons between clinical data among the groups were made using univariate analysis and χ^2^ test. The normality of the distributions of the discrete respiratory variables was ascertained using the Kolmogorov-Smirnov test. The occurrence of a positive noise limit in the airflow time series was compared using the χ^2^ test. Paired and unpaired T tests were used to study statistical differences of the linear and nonlinear measures of the inspiratory and expiratory flows among the groups. Pearson’s correlation coefficient was estimate for identifying significant relationships between airflow complexity and AlFO, FEV1/FVC, PaO_2_, PaCO_2_. Among the variables that had significant correlation with airflow complexity in univariate analysis, we then performed a multiple linear regression analysis to study the strength of its relation. During inspiratory load, the correlations were established in the whole population between airflow complexity and the mean negative BOLD signal.

### Mathematical Model of Respiratory Rhythmogenesis

Two pacemaker-like neurons have been identified in mammals in the ventro-lateral column of the brainstem, the pre-Bötzinger complex inspiratory group and parafacial expiratory group respectively [5]–[13]. Previous works showed that the parafacial group exhibits pre-inspiratory activity [9], [35] as well as a rebound bursting after inspiration [35] while the dynamics of both pacemakers display chaotic spike-bursting oscillations [14]. We therefore chose to develop a map-based model for respiratory rhythmogenesis for its relative simplicity compared with Hodgkin-Huxley formalism, and for its ability to generate spontaneous chaotic bursting activity [28], [29]. The model is developed based on the discrete version of FitzHugh-Nagumo model by adding Heaviside step function *H(x)*. Each pacemaker is modeled by the two dimensional original Courbage-Nekorkin map [28], [29] which is further refined to incorporate post-inhibitory rebound bursting behavior:
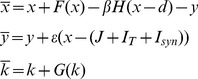
(1)Where *x* qualitatively defines the dynamics of the membrane potential of the neuron and *y* is the common variable specifying the dynamics of all outwards ionic currents (recovery variable). *β* and *d* controls the threshold properties of the oscillation, ε is a positive parameter setting the time scale of the recovery variable *y*. *J* is associated with excitability properties of the neuron; *F(x)* is a piece-wise linear version of the cubic function in the FitzHugh-Nagumo model:



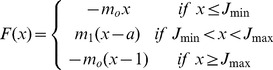
with 
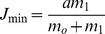
, 
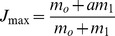
, and *m*_o_, *m_1_*>0

I_T_ is a low-threshold calcium Ca^2+^ current [67] defines as:
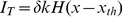
(2)Where *k* in equation (2) is a slow variable representing the inactivation of the low-threshold calcium conductance, which involves T-type Ca^2+^ calcium channels and produces a transmembrane current *I*_T_. δ represents the maximum conductance associated with *I_T_. G(k)* represents the dynamics of *I_T_* as follow:



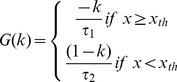
(3)In this form the model is capable of post-inhibitory rebound bursting when *x*_th_ is below the resting values of x. In equation (3), τ_1_ sets the duration of the burst and τ_2_ sets the duration of the hyperpolarization necessary to recruit a maximal post-inhibitory rebound response.

In equation (1), *I_syn_* is the chemical synaptic coupling between the pre-Bötzinger complex and the parafacial group in the following form:
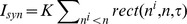
Where *K* is the coupling strength which value is positive for excitatory synapse and negative for inhibitory synapse and rect is the rectangle function as described below:



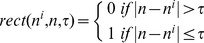
Where *n^i^* is the step of the ith spike in the presynaptic neuron and τ is the duration of the postsynaptic current. A post-inspiratory inhibitory feedback is introduced from the pFRG with the same amplitude and duration of the rebound bursts for “inspiratory off-switch” to prevent the preBötC from reactivation.

#### Modeling fMRI signal based on simulated neural activity

To confirm our hypotheses on different synchronization regimes of respiratory rhythmogenesis in healthy subjects and patients with respiratory failure, we performed 5 runs of simulations and then convolved the simulated neural states of the pre-Bötzinger complex and parafacial group with a hemodynamic response function. We used Statistical Parametrics Mapping software for the hemodynamic convolution: http://www.fil.ion.ucl.ac.uk/spm.

Under linear assumption, fMRI signals *m(t)* can then be modeled as a result of the convolution of neural states *s(t)* with a hemodynamic response function *h(t), ε(t)*is the noise.
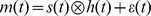
Where t is the time and ∶ denotes convolution, *h(t)* is the hemodynamic response function which is a mixture of two gamma functions. The parameter values of the hemodynamic response function are: delay of response relative to onset : 6 (s), delay of undershoot relative to onset = 16 (s), dispersion of response = 1 (s), dispersion of undershoot = 1 (s), ratio of response to undershoot 6 (s), onset = 0, length of kernel = 32 (s); ε(t) is the noise in the measurement assumed to be Gaussian white noise with mean zero and standard deviation 0.25. This value was chosen equal to the standard deviation of the mean BOLD time series. We model fMRI signal for 2 network schemes shown in figure 8 (B,C) and compute the AlFO of the modeled fMRI signal.

## Supporting Information

Figure S1 **End-tidal P_CO2_ measurements during unloaded and inspiratory resistive load (ventilatory flow measurements) as well as during fMRI acquisition.** Results are given for the 25 healthy subjects (A) and 25 COPD patients (B). C: End-tidal P_CO2_ measurements during resting state fMRI acquisition in healthy subjects (blue) and COPD patients (red). The means and standard deviations of the healthy subjects (n = 16) and the COPD patients (n = 17) are shown.
(ZIP)

Figure S2 **Comparisons between COPD patients having mild, moderate and severe dyspnea (Borg scale) at rest according to expiratory flow complexity (A), the amplitude of the low frequency oscillations (AlFO) of the ventro-lateral (VL) pons (B), and the pulmonary function index (FEV1/FVC) (C).** The patients with a severe dyspnea have a higher level of expiratory flow complexity and greater activity of the VL pons, as compared with patients having mild dyspnea. This difference is even less sensitive for the pulmonary function.
(ZIP)

Figure S3 **Linear correlation between expiratory flow complexity (top: Noise limit, bottom: Largest Lyapunov exponent) and pulmonary function index (FEV1/FVC) in the whole population of healthy subjects and COPD patients.** COPD patients are classified according to the diminution of their pulmonary function (GOLD classification).
(ZIP)

Figure S4 **Negative BOLD signal of the cerebral fMRI during inspiratory resistive loading in healthy subjects (left) and COPD patients (right).** Group analyses of the block design are given for the healthy subjects (left, n = 16) and COPD patients (right, n = 17). Sagittal and axial slices are shown on the top panel. Bottom: The corresponding mean time series of the ventro-lateral medulla of the 16 healthy subjects and 17 COPD patients are shown. The figures show the diminution of the BOLD signal during each application of the resistive load (5 cycles of rest (R: black line) and active task (A: red line) with resistive load).
(ZIP)

Table S1 Clinical characteristics of the 25 COPD patients.
(DOCX)

Table S2 Linear measures of the ventilatory variables (mean values, coefficients of variation and autocorrelation) during unloaded breathing and during inspiratory resistive load (20 cmH_2_0/L/sec).
(DOCX)

Table S3 Number of time series that exhibit positive noise limit value for chaos characterization in the inspiratory and expiratory flow time series in controls and COPD patients.
(DOCX)

Table S4 Summary of the results concerning airflow complexity and brainstem respiratory centers activity in healthy subjects and patients with chronic obstructive pulmonary disease (COPD).
(DOCX)
