# Supplementary figures and images for: Neural Mechanisms Underlying Breathing Complexity
Source: PLoS One. 2013 Oct 3;8(10):e75740. doi: 10.1371/journal.pone.0075740 (PMC3789752; doi:10.1371/journal.pone.0075740)

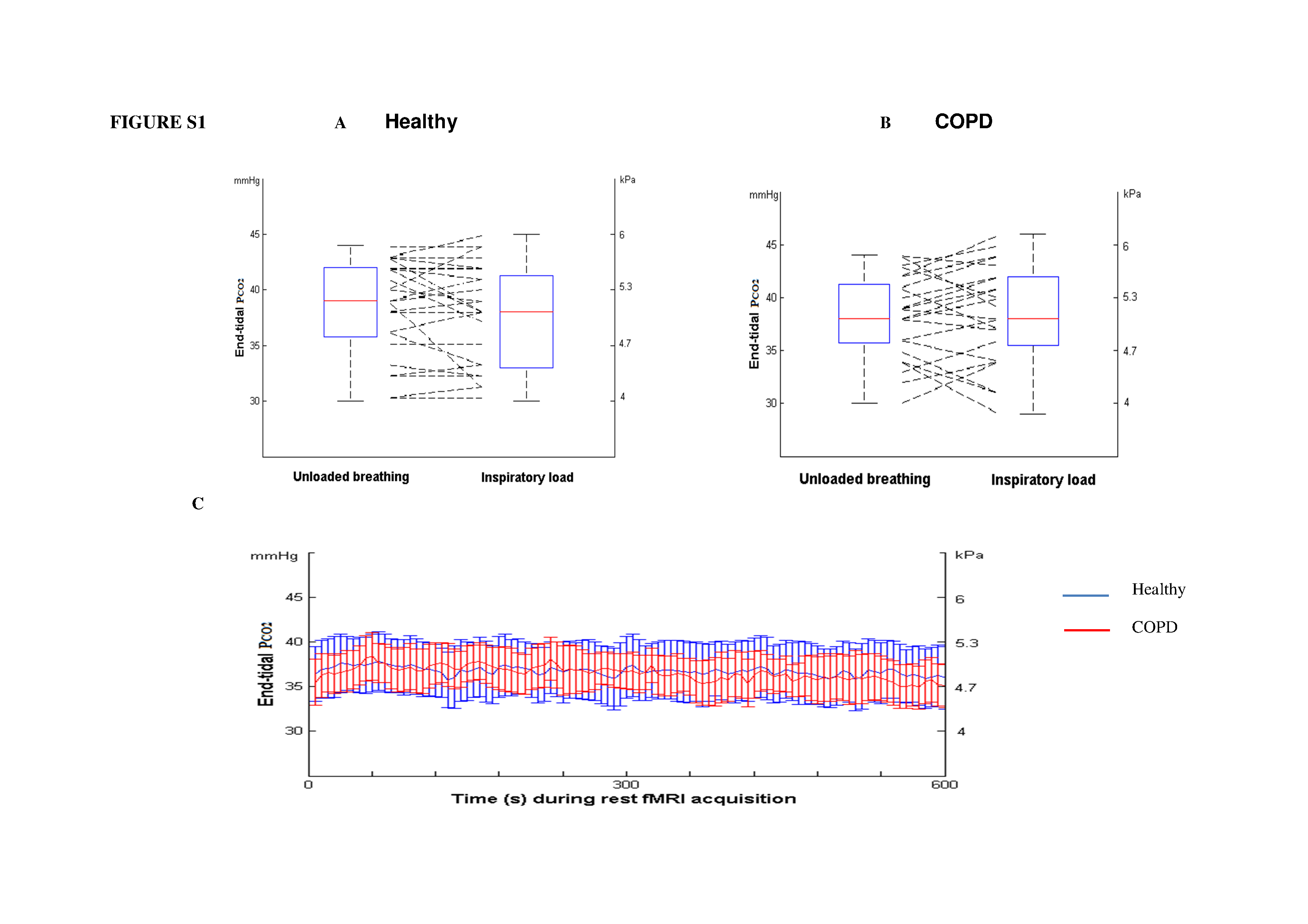

Supplement: Figure S1 — End-tidal PCO2 measurements during unloaded and inspiratory resistive load (ventilatory flow measurements) as well as during fMRI acquisition. Results are given for the 25 healthy subjects (A) and 25 COPD patients (B). C: End-tidal PCO2 measurements during resting state fMRI acquisition in healthy subjects (blue) and COPD patients (red). The means and standard deviations of the healthy subjects (n = 16) and the COPD patients (n = 17) are shown. (ZIP) [file pone.0075740.s001.zip › FIGURES1.tif]

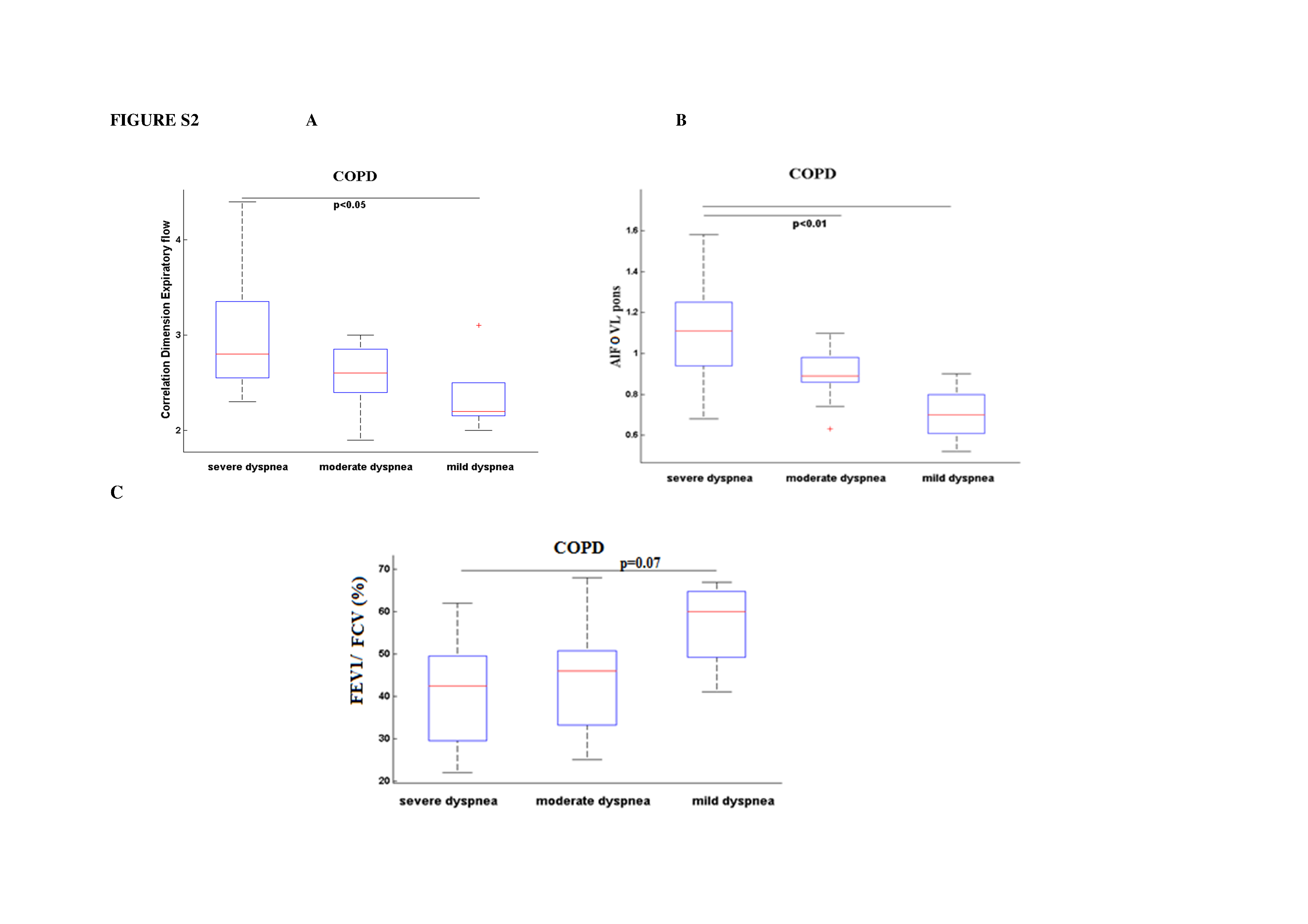

Supplement: Figure S2 — Comparisons between COPD patients having mild, moderate and severe dyspnea (Borg scale) at rest according to expiratory flow complexity (A), the amplitude of the low frequency oscillations (AlFO) of the ventro-lateral (VL) pons (B), and the pulmonary function index (FEV1/FVC) (C). The patients with a severe dyspnea have a higher level of expiratory flow complexity and greater activity of the VL pons, as compared with patients having mild dyspnea. This difference is even less sensitive for the pulmonary function. (ZIP) [file pone.0075740.s002.zip › FIGURES2.tif]

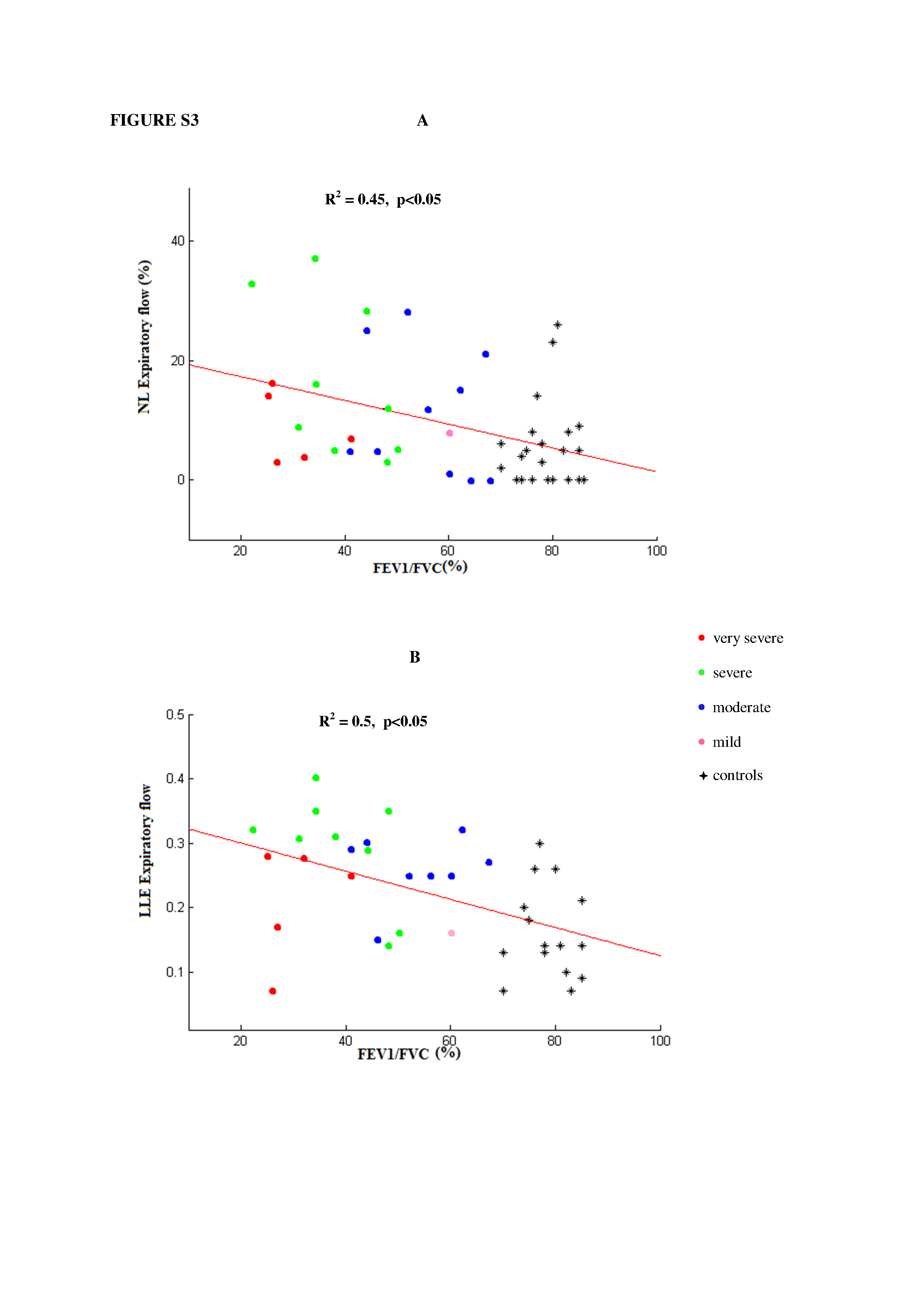

Supplement: Figure S3 — Linear correlation between expiratory flow complexity (top: Noise limit, bottom: Largest Lyapunov exponent) and pulmonary function index (FEV1/FVC) in the whole population of healthy subjects and COPD patients. COPD patients are classified according to the diminution of their pulmonary function (GOLD classification). (ZIP) [file pone.0075740.s003.zip › FIGURES3.tif]

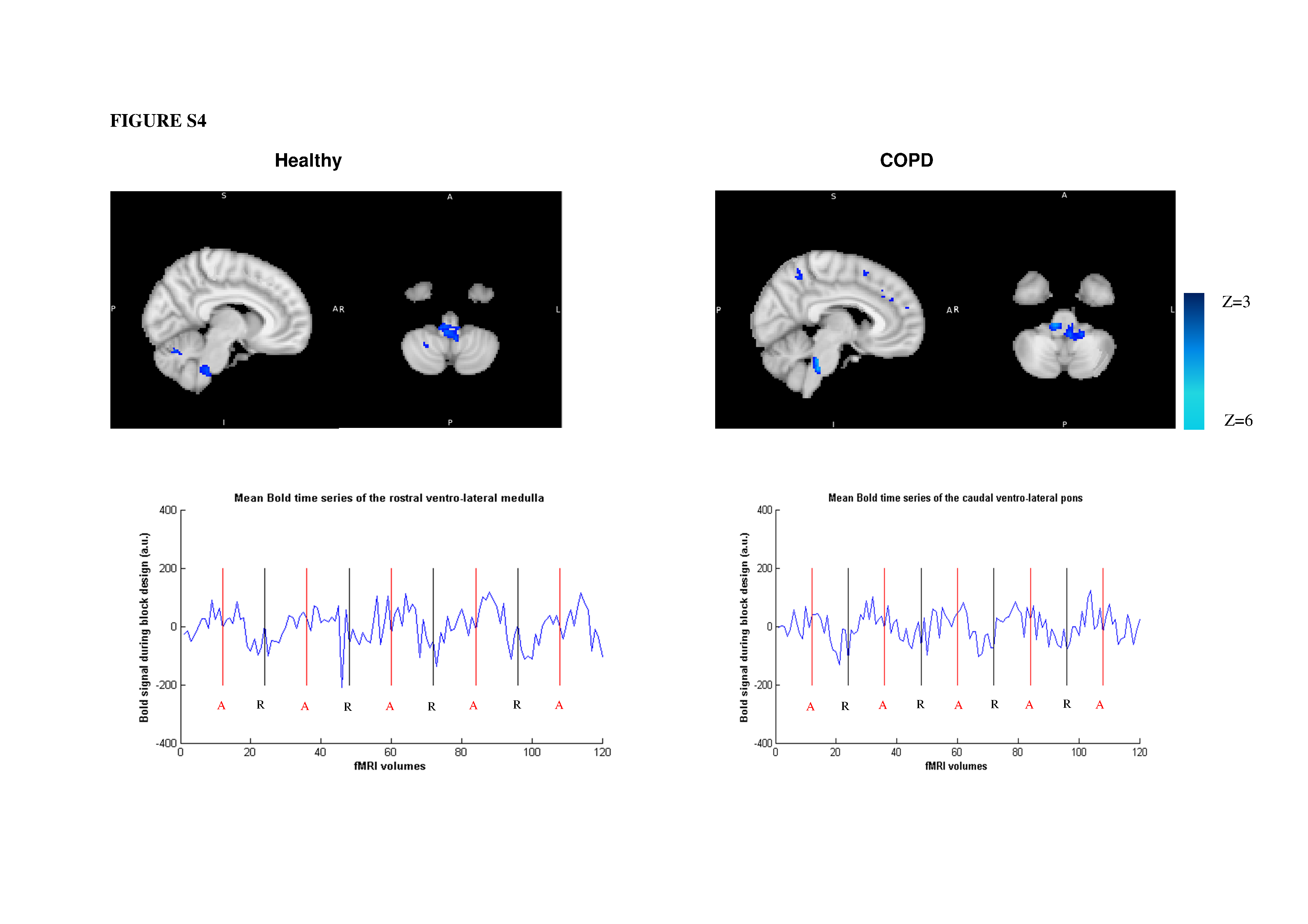

Supplement: Figure S4 — Negative BOLD signal of the cerebral fMRI during inspiratory resistive loading in healthy subjects (left) and COPD patients (right). Group analyses of the block design are given for the healthy subjects (left, n = 16) and COPD patients (right, n = 17). Sagittal and axial slices are shown on the top panel. Bottom: The corresponding mean time series of the ventro-lateral medulla of the 16 healthy subjects and 17 COPD patients are shown. The figures show the diminution of the BOLD signal during each application of the resistive load (5 cycles of rest (R: black line) and active task (A: red line) with resistive load). (ZIP) [file pone.0075740.s004.zip › FIGURES4.tif]
