# Supplementary material for: Neural Mechanisms Underlying Breathing Complexity
Source: PLoS One. 2013 Oct 3;8(10):e75740. doi: 10.1371/journal.pone.0075740 (PMC3789752; doi:10.1371/journal.pone.0075740)
Supplement: Table S1 — Clinical characteristics of the 25 COPD patients. (DOCX) [file pone.0075740.s005.docx]

**TABLE S1. Clinical characteristics of the 25 COPD patients**

| **Patients** | **Origin of disease** | **Severity (Gold)** | **FEV1/FVC (%)** | **FEV1 (%)** | **TLC (%)** | **RV (%)** | **P_O2_ (kPa /mmHg)** | **P_CO2_ (kPa/mmHg)** |
| --- | --- | --- | --- | --- | --- | --- | --- | --- |
| **1** | COPD | Moderate | 52 | 62 | 141 | 213 | 11.6 / 87 | 5.1 / 38 |
| **2** | COPD | Moderate | 67 | 79 | 101 | 128 | 12.7 / 95 | 5.2 / 39 |
| **3** | COPD | Moderate | 68 | 78 | 120 | 124 | 11.3 / 85 | 5.3 / 40 |
| **4** | COPD | Severe | 44 | 47 | 140 | 216 | 9.9 / 74 | 5.7 / 43 |
| **5** | COPD | Severe | 48 | 40 | 165 | 172 | 8.1 / 61 | 4.8 / 36 |
| **6** | COPD | Severe | 50 | 49 | 110 | 146 | 11.9 / 89 | 3.9 / 29 |
| **7** | COPD | Moderate | 64 | 69 | 90 | 94 | 12 / 90 | 5.3 / 40 |
| **8** | COPD | Moderate | 60 | 78 | 125 | 158 | 9.9 / 74 | 5.1 / 38 |
| **9** | COPD | Moderate | 56 | 79 | 95 | 113 | 12.1 / 91 | 5.1 / 38 |
| **10** | COPD | Moderate | 46 | 54 | 96 | 122 | 11.1 / 83 | 5.3 / 40 |
| **11** | COPD | Moderate | 62 | 78 | 141 | 195 | 11.6 / 87 | 5.3 / 40 |
| **12** | COPD | Severe | 34 | 42 | 100 | 163 | 10.8 / 81 | 5.5 / 41 |
| **13** | COPD | Severe | 31 | 40 | 150 | 287 | 8 / 60 | 5.1 / 38 |
| **14** | COPD | Moderate | 44 | 67 | 110 | 146 | 9.9 / 74 | 4.9 / 37 |
| **15** | COPD | Severe | 34 | 47 | 115 | 149 | 9.5 / 71 | 6 / 45 |
| **16** | COPD | Very severe | 27 | 26 | 102 | 228 | 8.8 / 66 | 5.6 / 42 |
| **17** | COPD | Mild | 60 | 97 | 120 | 150 | 9.9 / 74 | 5.3 / 40 |
| **18** | COPD | Severe | 38 | 38 | 105 | 185 | 11.1 / 83 | 4.5 / 34 |
| **19** | COPD | Moderate | 41 | 55 | 119 | 145 | 9.6 / 72 | 4.8 / 36 |
| **20** | Emphys. α1 AD | Very severe | 25 | 28 | 115 | 204 | 8.5 / 64 | 4.4 / 33 |
| **21** | Emphys. α1 AD | Very severe | 26 | 29 | 160 | 281 | 7.8 / 59 | 5.2 / 39 |
| **22** | Emphys. α1 AD  nn deficit | Very severe | 32 | 25 | 140 | 239 | 9.6 / 72 | 4.5 / 34 |
| **23** | Emphys. α1 AD | Very severe | 41 | 20 | 155 | 306 | 8.9 / 67 | 5.7 / 43 |
| **24** | Emphys. α1 AD | Severe | 48 | 48 | 112 | 195 | 8.8 / 66 | 4.7 / 35 |
| **25** | Emphys. α1 AD | Severe | 22 | 38 | 130 | 189 | 8.7 / 65 | 4.8 / 36 |

Emphys.: Emphysema; AD: anti-trypsin deficiency; FEV1: Forced expiratory volume in 1 sec; FVC: Forced vital capacity; TLC: Total lung capacity; RV: Residual volume
