# Supplementary material for: Neural Mechanisms Underlying Breathing Complexity
Source: PLoS One. 2013 Oct 3;8(10):e75740. doi: 10.1371/journal.pone.0075740 (PMC3789752; doi:10.1371/journal.pone.0075740)
Supplement: Table S2 — Linear measures of the ventilatory variables (mean values, coefficients of variation and autocorrelation) during unloaded breathing and during inspiratory resistive load (20 cmH20/L/sec). (DOCX) [file pone.0075740.s006.docx]

**TABLE S2. Linear measures of the ventilatory variables (mean values, coefficients of variation and autocorrelation) during unloaded breathing and during inspiratory resistive load (20cmH_2_0/L/sec).**

|  | **Controls (n=25)** | | | **COPD (n=25)** | | | **Controls vs COPD** | |
| --- | --- | --- | --- | --- | --- | --- | --- | --- |
|  | **L_0_** | **L_20_** | *pvalue L_20_ vs L_0_* | **L_0_** | **L_20_** | *pvalue L_20_ vs L_0_* | *pvalue L_0_* | *pvalue L_20_* |
| **Mean values** |  |  |  |  |  |  |  |  |
| **Ttot (s)** | 5.2±1.4 | 6.9±2.5 | *p=0.07* | 4.6±1.5 | 5.7±2.5 | *p=0.04* | *p=0.001* | *NS* |
| **Vt/Ti (l/s)** | 0.2±0.05 | 0.1±0.04 | *p<0.001* | 0.3±0.07 | 0.2±0.07 | *p<0.001* | *p<0.001* | *p<0.001* |
| **Vt/Te** | 0.21±0.05 | 0.2±0.07 | *NS* | 0.23±0.06 | 0.3±0.07 | *p=0.02* | *p=0.02* | *p=0.03* |
| **Coeff. of variation** |  |  |  |  |  |  |  |  |
| **Vt/Ti (l/s)** | 0.16±0.05 | 0.19±0.06 | *p=0.05* | 0.13±0.04 | 0.2±0.07 | *p=0.02* | *p=0.02* | *p=NS* |
| **Vt/Te** | 0.14±0.04 | 0.16±0.04 | *p=0.08* | 0.15±0.08 | 0.15±0.05 | *p=NS* | *p=NS* | *p=NS* |
| *pvalue Vt/Ti vs Vt/te* | *p<0.001* | *p=0.06* |  | *p=0.06* | *p=NS* |  |  |  |
| **Autocorr. one breath lag** |  |  |  |  |  |  |  |  |
| **Vt/Ti** | 0.7±0.15 | 0.85±0.12 | *p<0.001* | 0.6±0.1 | 0.7±0.2 | *p<0.001* | *p<0.01* | *p=0.03* |
| **Vt/Te** | 0.85±0.07 | 0.9±0.08 | *p<0.05* | 0.7±0.1 | 0.8±0.2 | *p=0.02* | *p<0.001* | *p=0.07* |
| *pvalue Vt/Ti vs Vt/te* | *p<0.001* | *NS* |  | *p<0.001* | *p=0.05* |  |  |  |

*Ttot: Total cycle time, Vt/Ti: inspiratory flow, Vt/Te: expiratory flow, Coeff.: coefficient, Autocorr.: autocorrelation*
