# Supplementary material for: Neural Mechanisms Underlying Breathing Complexity
Source: PLoS One. 2013 Oct 3;8(10):e75740. doi: 10.1371/journal.pone.0075740 (PMC3789752; doi:10.1371/journal.pone.0075740)
Supplement: Table S3 — Number of time series that exhibit positive noise limit value for chaos characterization in the inspiratory and expiratory flow time series in controls and COPD patients. (DOCX) [file pone.0075740.s007.docx]

**TABLE S3. Number of time series that exhibit positive noise limit value for chaos characterization in the inspiratory and expiratory flow time series in controls and COPD patients.**

|  | **Controls (n=25)** | | | **COPD (n=25)** | | | **Controls vs COPD** | |
| --- | --- | --- | --- | --- | --- | --- | --- | --- |
| **Time series** | **L_0_** | **L_20_** | *pvalue L_20_ vs L_0_* | **L_0_** | **L_20_** | *pvalue L_20_ vs L_0_* | *pvalue L_0_* | *pvalue L_20_* |
| **Inspiratory flow** | 17/25 | 6/25 | *p<0.01* | 20/25 | 10/25 | *p<0.01* | *p=NS* | *p=NS* |
| **Expiratory flow** | 15/25 | 8/25 | *p=0.09* | 25/25 | 16/25 | *p=0.04* | *p=0.001* | *p=0.05* |
| *pvalue Vt/Ti vs Vt/te* | *p=NS* | *p=NS* |  | *p=0.06* | *p=NS* |  |  |  |

*p values for* ***χ2*** *test are given.* ***L_0_*** *no load;* ***L_20_*** *inspiratory resistive load*
