# Supplementary material for: Neural Mechanisms Underlying Breathing Complexity
Source: PLoS One. 2013 Oct 3;8(10):e75740. doi: 10.1371/journal.pone.0075740 (PMC3789752; doi:10.1371/journal.pone.0075740)
Supplement: Table S4 — Summary of the results concerning airflow complexity and brainstem respiratory centers activity in healthy subjects and patients with chronic obstructive pulmonary disease (COPD). (DOCX) [file pone.0075740.s008.docx]

**TABLE S4. Summary of the results concerning airflow complexity and brainstem respiratory centers activity in healthy subjects and patients with chronic obstructive pulmonary disease (COPD).**

|  | **CHAOS** |  | **Brainstem BOLD signal** |  |
| --- | --- | --- | --- | --- |
|  | **Inspiration** | **Expiration** | **AlFO VL medulla (PreBötC)** | **AlFO VL pons (parafacial group)** |
| ***Unloaded breathing*** |  |  |  |  |
| **Healthy subjects** | **→** | **↓** | **→** | **↓** |
| **COPD** | ↑ | ↑↑ | **→** | ↑ |
| ***Loading Inspiration*** |  |  |  |  |
| **Healthy subjects** | **↓** | **→** | **↓** | **→** |
| **COPD** | **↓** | **↓** | **↓** | **↓** |

AlFO amplitude of the low frequency oscillations; VL: ventro-lateral; PreBötC: Pre-Bötzinger Complex
